# Development and Feasibility Assessment of an Intrinsic Capacity Program in Primary Care: Protocol for an Implementation Science Approach

**DOI:** 10.2196/84257

**Published:** 2026-02-02

**Authors:** Qing Wang, Mimaika Luluina Ginting, Ezra Ho, Siew Fong Goh, Jonathan Gao, Woan Shin Tan, Yew Yoong Ding, Wei Liang David Ng, Jonathan Choon Aik Ng, Sing Cheer Kwek, Richard Jor Yeong Hui, Zhen Sinead Wang, Chirk Jenn Ng, Grace Sum, The IMPACTFrail Group

**Affiliations:** 1 Geriatric Education & Research Institute Singapore Singapore; 2 Department of Geriatric Medicine Tan Tock Seng Hospital Singapore Singapore; 3 Lee Kong Chian School of Medicine Nanyang Technological University Singapore Singapore; 4 National Healthcare Group Polyclinics Singapore Singapore; 5 National University Polyclinics Singapore Singapore; 6 SingHealth Polyclinics Singapore Singapore; 7 Duke-NUS Medical School Singapore Singapore; 8 See Acknowledgments

**Keywords:** aged, world health organization, WHO, integrated care for older people, ICOPE, intrinsic capacity, frailty, feasibility, co-development, implementation science, primary care

## Abstract

**Background:**

The World Health Organization (WHO) public health framework for healthy aging advocates for action on the trajectories of intrinsic capacity (IC) across a person’s life course to optimize functional ability. While the WHO integrated care for older people (ICOPE) framework provides guidance on a systematic care pathway on IC screening, clinical assessment to clarify IC deficits and person-centered management, its real-world implementation and evaluation remain nascent. The Intrinsic Capacity Promotion in Primary Care for the Frail (IMPACTFrail) program for mildly frail older adults in Singapore’s primary care seeks to operationalize WHO ICOPE and national strategies.

**Objective:**

The objectives of this study are (1) the co-development of IMPACTFrail’s core functions and its delivery, as well as selecting, specifying and operationalizing implementation strategies to address anticipated barriers and leverage anticipated facilitators and (2) to conduct a feasibility assessment on the readiness to scale to a main study.

**Methods:**

For the first objective, the co-development process is guided by the United Kingdom Medical Research Council’s (MRC’s) framework for developing and evaluating complex interventions and the Framework of Actions for Intervention Development (FAID). The identification of contextual barriers and facilitators will draw on the updated Consolidated Framework for Implementation Research (CFIR) and its Outcomes Addendum. To identify individual-level behavior change barriers, we will extend this framework using the Theoretical Domains Framework (TDF). The Expert Recommendations for Implementing Change (ERIC) taxonomy guided our selection and development of implementation strategies. The collaboration involves implementation researchers, clinic leadership, frontline health care providers, and older adults. A 12-month, single-arm feasibility study will recruit 180 older adults aged 60 years and older with mild frailty (Clinical Frailty Scale score 4-5) across 5 public primary care clinics. Feasibility criteria include implementation, acceptability, practicality, and adaptability. We will narratively triangulate findings across study components to enhance the validity and credibility of the feasibility study, including (1) process evaluation using quantitative process indicators, (2) qualitative study to elicit barriers and facilitators to feasibility, sustainability and scalability, and to assess the attribution of the selected implementation strategies to implementation outcomes, (3) cost analysis, and (4) program description.

**Results:**

The study was funded in September 2024. Data collection for the feasibility assessment commenced in April 2025 and will conclude by March 2026. As of manuscript submission, 98 participants have been recruited across 5 sites. Recruitment, data collection, and analysis are ongoing. Publication of results is expected in early 2027.

**Conclusions:**

This protocol contributes to the literature by providing a detailed protocol on the co-development and feasibility testing of a complex intervention to enhance transparency, fidelity, and replicability. It disseminates knowledge on the integration of frameworks and methodologies to accelerate the translation of evidence to sustainable and scalable programs in practice.

**Trial Registration:**

ClinicalTrials.gov NCT06753643; https://clinicaltrials.gov/study/NCT06753643

**International Registered Report Identifier (IRRID):**

DERR1-10.2196/84257

## Introduction

### Background

The World Health Organization (WHO) conceptualizes healthy aging as the “ongoing process of developing and maintaining functional ability that enables well-being in older age” [1]. Intrinsic capacity (IC) refers to an individual’s combined physical and mental capacities (locomotion, vision, hearing, vitality, cognition, and psychological), while functional ability is the interaction between IC and the environment [1,2]. Recent literature reveals that IC is a predictor of adverse health outcomes, including functional decline, disability, hospitalizations, and mortality [3-6]. In response, the WHO public health framework for healthy aging advocates for action on trajectories of functional ability and IC across a person’s life course, particularly subgroups with stable or decreasing capacity prior to substantial losses [7].

To support the implementation of this approach, the WHO integrated care for older people (ICOPE) framework was introduced to provide guidance on optimizing IC and functional ability through person-centered care that prioritizes personal goals and needs. This reflects a shift from episodic and curative care to a holistic approach to healthy aging [8]. It promotes a systematic care pathway including Step 1 screening for IC deficits, Step 2 on assessments to clarify IC deficits, and Steps 3-5 on the development of personalized care plans, linkage to health and social care services to manage deficits, and engaging caregivers and communities for support [2]. Furthermore, IC is closely linked to frailty, the clinically recognizable state of increased vulnerability due to diminished physiological reserves in physical, cognitive, and social functioning [9,10]. Literature suggests that IC and frailty are interrelated constructs [11]. Declines in IC have been associated with incident frailty, and IC screening may enhance early detection and risk profiling [12-14]. Evidence also suggests that addressing IC declines could be more effective than exercise and nutritional interventions in prefrailty reversal to robustness [15].

In 2023, the Singapore Ministry of Health (MOH) published the National Frailty Strategy Report, which provides guidance on applying the WHO ICOPE framework to support mildly frail older adults in reversing frailty progression and optimizing functional ability [16]. This highlights the need to translate policy into practice. However, there is a dearth in the literature on the implementation and evaluation of the ICOPE in real-world settings [17,18]. International literature on early adopters from France and China identified several barriers. These include low adherence among older adults with more severe IC deficits, those who often need management the most [19]; anticipated challenges in scaling beyond feasibility study due to intense resource use [20]; and limited reach to older adults who are less technologically savvy when telehealth is used as the primary delivery mode [21]. While these findings are insightful, they may not be generalizable to Singapore due to the difference in health care systems and local cultures. IC screening can be implemented across various settings, including community and primary care. While recent local studies have assessed the anticipated determinants and feasibility of implementing IC screening in community settings, no studies have examined its implementation in primary care [18,22,23], which is often the first point of contact for patients accessing health care services [24]. To elaborate, existing health care programs that conduct IC screening are in active aging centers and community clubs to serve community-dwelling older adults in Singapore. These community-based centers are located in close proximity to public residential housing for ease of access [18,25]. They offer a range of services such as befriending services, workshops (eg, cooking and arts and crafts), exercise programs, and chronic condition health screenings. In contrast, there are no health care programs that detect and manage IC decline at public primary care clinics and private general practices in Singapore. Moreover, these studies do not specifically target mildly frail older adults, a group with greater potential for early screening and intervention to prevent further functional decline and a higher likelihood of reverting to robustness compared to their frail counterparts [26].

Additionally, it is common for complex interventions to fall short of achieving sustainment and scalability when implemented in real-world practice settings outside research environments [27,28]. ICOPE programs for community-dwelling older adults are inherently complex, involving multiple components, diverse stakeholders, and the need for integration with existing workflows and infrastructures. Hence, it is crucial to account for contextual factors, which are the dynamic forces for and against successful implementation across macro, meso, and individual levels [29]. In response, there is growing advocacy for systematic approaches to designing, implementing, and evaluating complex health care interventions, drawing on both complex intervention research and implementation science frameworks [27,30-32]. This study protocol outlines the co-development of an IC management program in Singapore and its feasibility assessment. The program’s development and feasibility assessment are guided by complex intervention research and implementation science frameworks, using a phased approach that includes development, feasibility testing, and scaling up for evaluation. This approach is designed to enhance implementation outcomes such as reach, adoption, fidelity, and sustainability. In the long run, it aims to support integration into routine practice, promote adoption by implementers, and improve uptake and adherence among older adults [30,31]. The reporting of this protocol is guided by the adapted SPIRIT (Standard Protocol Items: Recommendations for Interventional Trials) guideline (see Multimedia Appendix 1) [33,34].

### The Intrinsic Capacity Promotion in Primary Care for the Frail Program

**Figure 1:**
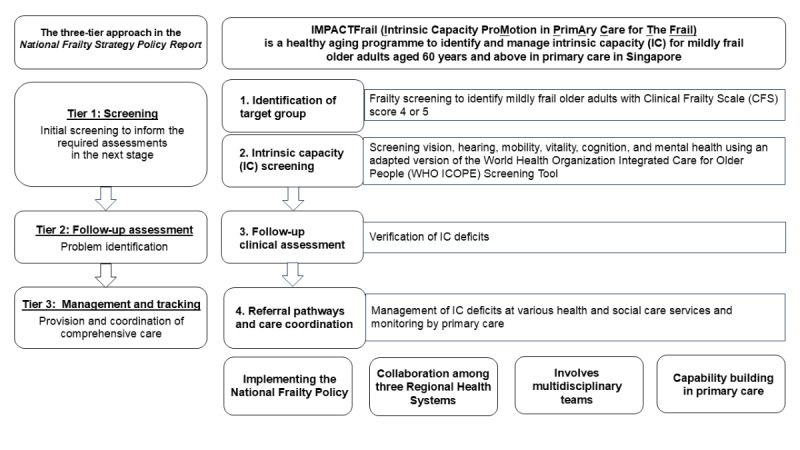
The 3-tier approach in the National Frailty Strategy Report and Intrinsic Capacity Promotion in Primary Care for the Frail (IMPACTFrail) care model.

Core functions of Intrinsic Capacity Promotion in Primary Care for the Frail (IMPACTFrail) are aligned with the WHO ICOPE framework and the 3-tier approach of the National Frailty Strategy (Figure 1):

1. IC screening will be conducted for older adults aged 60 years and older who are mildly frail, as assigned by a trained health care provider as Clinical Frailty Scale (CFS) score 4 or 5. The CFS was selected by MOH as a national community screening and segmentation tool [16,35]. It is a 9-point frailty screening tool that ranges from CFS 1 (very fit) to 9 (terminally ill) [35]. The policy proposes to target the CFS 4 (very mild frailty) and 5 (mild frailty) groups, as they are anticipated to benefit the most and to support optimal resource allocation. CFS 6-7 individuals (moderate to severe frailty) may benefit from directly undergoing comprehensive geriatric assessment (CGA) [16,36,37], and the CFS 8-9 group requires distinctly different care such as end-of-life support instead of community-based health services.
2. Follow-up clinical assessments to clarify IC deficits. This refers to postscreening assessment for IC deficits using clinical tools and scales by health care providers, such as the short physical performance battery for mobility or the Abbreviated Mental Test for cognition.
3. Establishment of care pathways to health and social care services to manage and monitor IC deficits and other identified needs. These individualized care plans will be overseen by the primary care physicians.

Grounded in the 3-tier approach, IMPACTFrail seeks to translate the National Frailty Strategy specifically to local primary care contexts. First, evidence on the implementation of the ICOPE framework in primary care is still limited and may not be generalizable to Singapore’s contexts in terms of health care system infrastructures, policies, and cultures [17,38]. Second, IMPACTFrail’s ICOPE Step 1 in primary care may complement early local efforts on IC screening via community-based programs at active aging centers and community care organizations [22,23]. A proportion of community-dwelling older adults do not participate in community-based programs, and public primary care clinics may play a strategic role in capturing these older adults. Primary care physicians have also been identified as the preferred personnel to coordinate personalized care, and IMPACTFrail may facilitate this relationship by delivering ICOPE Step 1 in primary care at the outset [39,40]. Third, existing primary care national initiatives such as Healthier SG, which promotes a “one patient, one family doctor” model for chronic condition management [41], are complemented by IMPACTFrail’s holistic focus on functional ability. IMPACTFrail is also aligned with Age Well SG, a national program on aging well in the community [42].

To facilitate sustainability and scalability, the development of IMPACTFrail involves public primary care clinics across Singapore’s 3 integrated health care systems from the outset (Figure 1). Briefly, Singapore’s health care system is structured into 3 integrated regional health systems. Primary care is provided by private general practitioners and 26 public clinics. While all 3 health care systems are under MOH, they have autonomy in carrying out their functions [43]. Each of the 3 systems is accountable for the health of their geographically defined populations. There is impetus to accelerate collaborative efforts among all 3 systems to deliver national initiatives cohesively [24]. Second, IMPACTFrail serves to strengthen capability building in primary care (Figure 1). Targeted training is conducted to equip multidisciplinary teams, including doctors, nurses, care coordinators, and allied health care workers (eg, physiotherapists and dieticians) with skills needed for CFS assignment, IC screening, and follow-up clinical assessments. Third, IMPACTFrail facilitates collaborations among multidisciplinary and interprofessional stakeholders. Buy-in from clinic leadership is required for integration of IMPACTFrail within each clinic’s ecosystem of programs and workflows. IMPACTFrail involves nonmedical staff, family doctors, family medicine specialists, nurses, and allied health professionals (eg, dieticians and therapists). The clinics build on partnerships with specialist outpatient clinics and community care organizations to manage older adults’ IC deficits.

### Study Aims and Hypotheses

The overall aim of the protocol is to describe the co-development and feasibility assessment of the implementation of a novel IC detection and management program, along with its implementation strategies, for mildly frail older adults in the primary care setting in Singapore. Specific objectives include:

- Objective 1: to collaboratively develop the form of delivery of IMPACTFrail’s core components, and select, specify and operationalize implementation strategies to mitigate anticipated barriers and leverage anticipated facilitators to implementation. The co-development involves implementation researchers as well as health care administrators and frontline health care providers from all three health care systems.
- Objective 2: to conduct a multisite feasibility study to assess the feasibility of implementing IMPACTFrail and its selected implementation strategies in contextualized settings. Specifically, we will conduct a process evaluation using quantitative process indicators, a qualitative study to elicit the barriers and facilitators to feasible, sustainable, and scalable implementation, a cost analysis to understand resources needed to develop and implement the program, and documentation of the program description and adaptations.

We anticipate the following:

- Hypothesis 1: we hypothesize a successful co-development of the program’s core components and implementation strategies. This is due to stakeholder engagement and collaboration with clinic leadership teams and frontline implementers from the outset.
- Hypothesis 2: overall, we hypothesize a moderate to high level of feasibility. This is due to the program being supported by the selection, specifying, and development of implementation strategies to mitigate anticipated barriers and leverage anticipated facilitators. We anticipate barriers at the macro, meso, and individual levels, given that IMPACTFrail is a complex intervention involving multiple components and stakeholders. At the patient level, we also anticipate barriers related to behavioral change, such as low motivation and limited opportunity, as older adults may lack health-seeking behaviors or support to follow through on postscreening assessment and interventions. Cost analysis may reveal marginally high resource use from manpower costs for the initial capability building of health care providers. Barriers to sustainability and scalability are anticipated due to competing resource demands and priorities in primary care.

## Methods

### Integration of Frameworks

Both objectives are guided by integrating complex intervention research and implementation science frameworks (Table 1). First, this study is guided by the overarching United Kingdom Medical Research Council’s (MRC’s) framework for developing and evaluating complex interventions [30]. IMPACTFrail is a complex intervention with multiple core functions, implementation strategies, and involvement of stakeholders (decision-makers, frontline implementers, and older adults). The MRC framework divides complex intervention research into four phases: (1) development and identification of the intervention, (2) feasibility testing, (3) larger-scale evaluation, and (4) implementation [30]. Phase 1 and 2 correspond to the development of IMPACTFrail and feasibility assessment, respectively. The latter 2 phases of the MRC framework—evaluation and implementation—will be conducted in subsequent studies. Second, Framework of Actions for Intervention Development (FAID) guides Objective 1 [44]. It serves as an extension to the MRC framework’s development and identification of an Intervention, and informs the key principles and actions for consideration when developing IMPACTFrail.

**Table 1:** Integration of frameworks for complex intervention research and implementation research.

Framework and guidelines	What it informs	Elaboration
Integration of the United Kingdom Medical Research Council (UK MRC) framework for developing and evaluating complex interventions [30] and the Framework of Actions for Intervention Development (FAID) [44]	Objectives 1 and 2	The MRC guidance is a high-level framework that connects the development process to subsequent pilot-testing, implementation, and evaluation. The FAID informs on the list of actions to undertake in the process of developing an intervention. It provides more detailed guidance on the MRC framework’s development stage.
Updated Consolidated Framework for Implementation Research (CFIR) and its Outcomes Addendum [31]	Objectives 1 and 2	Anchors the implementation research. Guides the understanding of contextual determinants to attaining successful implementation outcomes.
Integration of Updated CFIR [31], Expert Recommendations for Implementing Change (ERIC) taxonomy [32], and the Proctor framework for specifying and reporting strategies [45]	Objective 1: Study component on selecting, specifying, and developing implementation strategies	Informs the selection and specifying of implementation strategies to mitigate CFIR-identified barriers and leverage CFIR-identified facilitators.
Guidelines on conducting feasibility studies [30,46-48]	Objective 2	Guidelines inform the components of a feasibility study and assessment of feasibility criteria.
Proctor framework for specifying and reporting strategies [45]	Objective 2: Study component on assessing fidelity of implementation strategies	Monitor and track the fidelity of operationalizing the specified domains (actor, action, action target, dose, temporality) of each selected ERIC strategy using a comprehensive list of indicators.
Integration of Updated CFIR [31], the Theoretical domains framework (TDF) [49], and the ERIC taxonomy [32]	Objective 2: Qualitative study component	Expand CFIR’s Individual Characteristics domain by mapping determinants of Capability, Opportunity, and Motivation to TDF domains. ERIC taxonomy informs the refinement of existing implementation strategies and selection of new strategies.

Third, our study is anchored in the updated Consolidated Framework for Implementation Research (CFIR) and its Outcomes Addendum. The updated CFIR is a determinant framework that guides the understanding of contextual factors that influence successful implementation of complex interventions, while its Outcomes Addendum guides the evaluation of implementation outcomes such as adoption, implementation, sustainability and scalability [31]. CFIR is one of the most widely used implementation science frameworks and this study applies the updated version with 48 constructs across five domains (innovation, outer setting, inner setting, implementation process, and individuals). Additionally, we will apply the Theoretical Domains Framework (TDF) to expand on CFIR’s “Characteristics of Individuals” domain [49]. Specifically, the TDF expands on the Capability, Opportunity, Motivation, Behavior (COM-B) model, a behavior change framework that proposes three necessary components for any behavior to occur [49]. The Expert Recommendations for Implementing Change (ERIC) taxonomy and Proctor framework on reporting and specifying implementation strategies will also be used [32,45,50]. The application of these frameworks will be elaborated in the subsequent sections. Finally, published guidelines on conducting feasibility studies will inform the feasibility assessment [30,46-48].

### Objective 1: Co-Development of IMPACTFrail and Implementation Strategies (6 Months)

#### Aim

We aim to collaboratively develop (1) the forms of delivery of IMPACTFrail’s core components and (2) implementation strategies to support implementation. This collaboration will involve the research team, health care administrators (clinic leadership teams with decision-making authority), and frontline health care providers, including doctors, nurses, and allied health professionals.

#### Study Procedure

Prior to this study, the research team conducted 6 months of informal stakeholder engagement (total of 22 interviews and focus group) with the clinics’ leadership, frontline health care providers, and older adults. Overall, we aimed to explore their awareness of frailty and IC, elicit anticipated barriers and facilitators of implementing or participating in the program, and understand the contextual operational and logistical constraints at each clinic. Our study builds on the information from the stakeholder engagement. To elaborate, we conducted 3 interviews with 3 older adults who were existing patients of the clinics to elicit expected barriers to participating in IMPACTFrail and to provide suggestions to encourage older people to attend IC screening. We conducted 7 interviews with 8 individuals who held mid-to-senior management positions in the clinics’ leadership team and 5 interviews and 5 focus groups with family medicine doctors (n=10), nurses (n=10), allied health professionals (n=2), care coordinators who assist doctors and nurses (n=4), and nonmedical support staff (n=6). Health care professionals were across different levels of seniority and whom we anticipated to be involved across different steps of IMPACTFrail’s patient journey. Sampling was guided by using the principle of sufficiency [51,52]. Additionally, we interviewed 2 private general practitioners to gain some insights from the private sector on implementing an IC detection and management program in a primary care setting.

The individual or paired interviews were for health care administrators and older adults to encourage open sharing, particularly in light of potential power dynamics and different language and cultural backgrounds. Focus groups with multidisciplinary teams were to gather different perspectives on the feasibility of the program. Interviews with older adults were conducted in their preferred language, either English or Chinese, by multilingual research team members. Engagement sessions with health care administrators and frontline health care providers were conducted in English. The engagement aimed to explore participants’ awareness of frailty and IC, elicit anticipated barriers and facilitators to implementing or participating in the program, and understand the operational and logistical context at each clinic. The study design and implementation were informed by insights through informal stakeholder engagement and guided by complex intervention research and implementation science frameworks.

#### Co-Development of the Core Components

The core functions of IMPACTFrail (the “what”) are informed by Singapore’s National Frailty Strategy, while the forms of delivery (the “who” and “how”) will be collaboratively developed with stakeholders in this study. Examples of specific output from this co-development stage may include: (1) IC screening questions adapted from the original WHO ICOPE screening tool (see Multimedia Appendix 2) tailored to each clinic’s clinical protocols and preferences; (2) modifications to electronic medical record systems to capture new data required by IMPACTFrail; (3) clear patient journey pathways; (4) identification of relevant clinic staff (doctors, nurses, allied health professionals, clinic coordinators, and support staff) and delegation of roles; and (5) capability building plans to prepare for the upcoming feasibility study.

#### Co-Development of Implementation Strategies

We will select, specify, and develop implementation strategies to mitigate anticipated barriers and leverage anticipated facilitators to implementation. Implementation strategies are defined as “methods or techniques used to enhance the adoption, implementation, and sustainability of a clinical program or practice” [45]. As we will conduct a smaller scale feasibility study in the next stage, each clinic will only implement a tailored set of 2-5 implementation strategies. These implementation strategies could be from the list of the 73 ERIC strategies or created inductively [32]. Each clinic could select and develop a different set of implementation strategies to align with their specific contexts.

The anticipated barriers and facilitators identified during the informal stakeholder engagement will be mapped to the CFIR determinant constructs. Subsequently, we will use the CFIR-ERIC matching tool to match CFIR-identified determinants to ERIC strategies [53,54]. The research team, polyclinic leadership, and frontline health care providers will collaboratively review the identified barriers and facilitators, the output from the CFIR-ERIC matching tool, and local contextualized considerations (eg, clinic operations, manpower) to prioritize and select implementation strategies. Strategy selection will be guided by 5 literature-informed criteria: relevance, opportunity for improvement, feasibility, level of difficulty, and validity [55]. The selected implementation strategies will be specified using Proctor framework for reporting and specifying implementation strategies across 6 domains, including actor, action target, action, temporality, dose, and implementation outcomes [45], and subsequently developed for operationalization in the feasibility study [46,56].

### Objective 2: Multisite Feasibility Study (12 Months)

#### Overall Aim

The feasibility study is defined as a small-scale assessment aimed at determining whether IMPACTFrail can be implemented at this level, evaluating its potential for scalability, and identifying how it might be adapted for broader implementation [46,56]. In other words, we seek to evaluate the readiness of implementers delivering IMPACTFrail in real-world settings at a larger scale and the preparedness of older persons to participate in the program [30,47,48]. This protocol precedes a subsequent hybrid effectiveness-implementation study that will be conducted at a larger scale with more sites and older adult participants [57,58]. The study was registered on ClinicalTrials.gov on December 22, 2024 (Identifier: NCT06753643).

#### Study Design

This will be a single-arm feasibility study conducted at five public clinics across all 3 regional health systems in Singapore. Specifically, 2 clinics were selected from each of 2 regional health systems, and 1 from the third System. Each clinic has contextualized barriers and facilitators to implementation, clinic workflows, and infrastructure. Thus, each clinic is conceptualized as a single site for feasibility testing. This study does not include a comparator arm or a pre-post design, as it does not aim to assess effectiveness. Instead, it focuses on a small-scale feasibility assessment to inform the design and implementation of a subsequent, statistically powered hybrid effectiveness-implementation. The phased approach increases the likelihood of successful real-world implementation by enabling early refinement and adaptation in practice settings.

#### Eligibility Criteria and Sample Size

Eligibility criteria include (1) registered patient of the clinic, (2) aged 60 years and older (3 of 5 clinics) or aged 65 years and older (2 of 5 clinics), (3) mildly frail, assigned by a trained health care provider as CFS score 4 or 5, and (4) able to provide informed consent independently or through a legally authorized representative. Exclusion criteria include (1) administered comprehensive geriatric assessment in the past 12 months or (2) at the time of this writing, being under the care of a geriatrician or patient of a geriatric specialist outpatient clinic.

Older adults who meet these eligibility criteria will be given information on the IMPACTFrail health care program and the research study verbally and via a hardcopy brochure by a paraprofessional care coordinator or nurse. A trained research assistant will obtain written informed consent from each older adult individually and address any queries from potential participants. Consent-taking to be recruited as a participant of the IMPACTFrail study will be conducted prior to intrinsic capacity screening. Recruitment will be conducted over approximately 9 months, continuing until each clinic reaches its target of 36 participants. All older adults will only need to go through this process once. Additionally, a subset of these older adult study participants of IMPACTFrail who participate in in-depth interviews of the qualitative study will undergo a separate informed consent process before their interviews. This informed consent-taking will be facilitated by trained researchers.

This set of consistent and unified inclusion and exclusion criteria will facilitate meaningful comparison of findings across sites and generalizability. Importantly, the broad objective is to translate the National Frailty Strategy to real-world practice. To facilitate this, the intervention will be structured around core functions that are common across all 5 participating clinics, including Clinical Frailty Scale score assignment, IC screening for mildly frail older adults, further clinical assessment, tailored referrals to clinics, specialists or community services, and monitoring and follow-up. While the form of implementation may vary across clinics, such as the precise age cut-offs for inclusion, further clinical assessment tools, and referral workflows, the underlying core functions remain consistent and intentional. This approach is aligned with the core functions and forms paradigm, recognizing that complex interventions must adapt to context while preserving their core purpose [59-61].

For the 2 of 5 clinics that will apply a slightly higher age cut-off of 65 years (instead of 60 years) for inclusion, it serves to facilitate integration of IMPACTFrail’s frailty screening with existing health programs and clinical protocols. They anticipated operational challenges and a lack of manpower to intentionally identify older adults 60-64 years old for the feasibility study.

Additionally, 2 clinics under the same regional health system have additional inclusion criteria. These criteria include (1) enrolment in Healthier SG [56], (2) absence of dementia diagnosis, and (3) active chronic disease appointments for any one of the following chronic conditions: Type 2 diabetes mellitus, chronic ischemic heart disease, congestive cardiac failure, osteoporosis, Stage 4 chronic kidney disease, and chronic obstructive pulmonary disease. These criteria are driven by contextual operational considerations within these clinics, including manpower capacity, logistics, and the need to align with ongoing preventive and management care programs for patients with chronic conditions. This more targeted approach was assessed to be more acceptable and pragmatic for small-scale feasibility testing in settings with constrained resources, while keeping the same core frailty screening, IC screening, and management functions.

Overall, this study protocol serves the purpose of disseminating knowledge on co-developing a health care intervention and implementation strategies, with built-in flexibility to accommodate system-level differences across clinics. In line with implementation research, we will subsequently assess how clinics could gradually align in eligibility criteria and protocols as IMPACTFrail scales up nationally.

No formal sample size calculation is performed, as this is a feasibility study not designed to be statistically powered for assessing effectiveness and health outcomes. There is no consensus in the literature on the required sample size for pilot and feasibility studies [62]. Recommendations of sample size vary depending on the primary objective of the study, the feasibility parameter being evaluated, or clinical scale evaluation [63-66]. Published reviews from the United Kingdom have reported median sample sizes of 30 and 36 participants per arm in feasibility studies [67,68]. In addition, clinics leads were also consulted on the practical projections of recruitment and uptake, taking into account the study timeframe and available resources. Hence, our study aims to recruit 36 older adults per clinic. This was computed based on targeting a minimum of 30 participants per site while accounting for an anticipated 20% loss to follow-up (eg, nonattendance at follow-up assessment post screening). Each clinic is conceptualized as an individual site due to contextualized barriers, workflows, and infrastructure.

#### Feasibility Criteria

Table 2 describes the four feasibility assessment criteria: (1) implementation, (2) acceptability, (3) practicality, and (4) adaptability, along with the corresponding study components mapped to each criterion. The criteria are informed by the literature on feasibility studies [47]. In summary, we prioritize the need to assess the ability to carry out the new screening and integrating the core functions with existing clinical protocols (implementability), how a satisfying or attractive a new program that focuses on domains beyond the conventional chronic conditions is to health care providers and older people (acceptability), whether the resources can support such a new program (practicality), and the degree it can be improved or changed if we progress beyond the pilot (adaptability) [47]. The criteria collectively inform us on the feasibility to progress from the small-scale pilot study to a main study that will be statistically powered to evaluate effectiveness and implementation at scale.

**Table 2:** Mapping of study components to feasibility assessment criteria.

Criteria	Study components
Implementation: to assess how well the program’s core components and implementation strategies are implemented as intended.	Study component 1: quantitative process indicators Study component 2: assess fidelity of implementation strategies Study component 3: qualitative study Identify deviations or adaptations of the core components Elicit barriers and facilitators perceived by implementers and older adults on implementing or receiving the program and its implementation strategies Study component 5: program description Study component 6: logic model and theory of change
Acceptability: to assess the satisfaction and adoptability of the program’s core components and implementation strategies.	Study component 3: qualitative study to identify barriers and facilitators to acceptability, adoptability, and appropriateness perceived by implementers and older adults on the program and its implementation strategies
Practicality: to assess the ability to carry out the program’s core components and implementation strategies in a sustained manner.	Study component 3: qualitative study to identify barriers and facilitators to (1) delivering the program and implementation strategies with the given resources (eg, manpower, information technology infrastructure, physical infrastructure) and (2) sustainability Study component 4: cost analysis
Adaptability: to assess the extent that barriers to implementation, acceptability, adoptability, practicality, sustainability and scalability can be mitigated. This section includes assessing if the program and implementation strategies can be adapted and refined, and if we have the ability to develop additional implementation strategies if we scale.	Study component 3: qualitative study to gain insights on (1) adaptations to the delivery of the program’s core components and implementation strategies, to facilitate implementation, acceptability, adoptability, practicality, sustainability, and scalability and (2) selecting and developing additional implementation strategies if we scale

### Study Component 1: Quantitative Process Indicators on Care Processes

#### Aim

We will collect and document a comprehensive list of quantitative process indicators to measure the processes of care (Table 3). This addresses the research question on whether the program activities are delivered as planned and informs us on the follow-through of patients for the entire patient journey.

**Table 3:** Process indicators.

Process indicators			Measure	Proctor implementation outcomes
**Patient recruitment**				
		Number and proportion of older adults screened for frailty using the Clinical Frailty Scale (CFS)	Volume of older adults screened for frailty using the CFS	Reach Adoption
		Number and proportion of older adults screened as CFS 4-5	Volume of older adults with mild-to-moderate frailty (CFS 4-5)	Reach Adoption
		Number and proportion of CFS 4-5 older adults referred to IC screening	Volume of older adults directed to secondary IC screening	Reach Adoption
**Intrinsic Capacity (IC) screening for CFS 4-5**				
		Number and proportion of older adults who initiated IC screening	Receptivity to IC screening	Reach Adoption
		Time between CFS screening to initiation of IC screening (in days)	Efficiency of initiating IC screening after CFS segmentation	Reach Adoption
		Number and proportion of older adults who completed all 6 domains of IC screening	Completion of IC screening after it was initiated	Reach Adoption
		Number and proportion of older adults that completed each IC domain	Completion rates of each IC screening domain	Acceptability
		Duration between initiation of IC screening to completion of IC screening of 6 domains (in days or weeks)	Efficiency of completing all 6 domains of IC screening after initiation of IC screening. For example, IC screening of 6 domains may be staggered across two visits. This indicator measures the duration between the screening of at least 1 IC domain to the completion of all 6 IC domains	Fidelity
**Follow-up assessment**				
		Number and proportion of referrals to follow-up assessment for each IC deficit	This measures care coordination: (1) number of referrals made to services for follow-up assessment for each IC deficit and (2) proportion of referrals to each service with the denominator as the total number of referrals	Reach Adoption Penetration Fidelity
		Number and proportion of actualized referrals to follow-up assessment for each IC deficit	This measures care coordination: (1) number of actualized referrals to services for follow-up assessment for each IC deficit and (2) proportion of actualized referrals to each service with the denominator as the total number of referrals	Reach Acceptability Costs
**Downstream care pathways**				
	Number and proportion of referrals for the management of each IC deficit		This measures care coordination: (1) number of referrals made to services for management of each IC deficit and (2) proportion of referrals to each service with the denominator as the total number of referrals	Adoption Penetration Fidelity
	Number and proportion of actualized referrals for the management of each IC deficit		This measures care coordination: (1) number of actualized referrals to services for management of each IC deficit and (2) proportion of actualized referrals to each service with the denominator as the total number of referrals	Reach Penetration Costs
**Capability building**				
		Number of staff trained to conduct CFS and IC screening, and to provide referrals to follow-up assessments and care pathways Include breakdown by occupation (doctor, nurse, care coordinator, and patient service associate)	Capability building for screening and delivery of referrals	Adoption Feasibility Acceptability Costs

#### Study Sample and Data Source

The sample includes all recruited older adult participants. Each of the 5 clinics will collect and provide data to the research team. Table 3 summarizes the list of process indicators.

### Study Component 2: Fidelity Indicators for Implementation Strategies

#### Aim

We aim to assess the fidelity of the selected implementation strategies.

#### Study Procedure

We will monitor and track the fidelity of operationalizing each selected ERIC implementation strategy across the specified domains (actor, action, action target, dose, or temporality), using a comprehensive set of indicators [45]. In other words, we will assess whether each strategy is delivered in the feasibility study as originally intended during the co-development phase.

Indicators will be collaboratively designed by the research team and clinic implementation leads. Data will be collected regularly by clinic implementation leads and frontline implementers. The research team will systematically document any deviations from the planned implementation and discuss them with clinic implementation leads. Adaptations to strategies, if any, will also be documented. For example, in the case of the implementation strategy involved educational sessions for capability building on IC screening, we will assess whether the planned elements, such as the trainers (actor), attendees (target), and the frequency and duration of training (dose), are delivered as intended. If discrepancies arise, we will explore whether fidelity can be improved or whether adaptations are needed. In addition, we will focus on sustainability and scalability, such as whether the frequency and dose of the strategies are sustainable within the clinic and adaptable for scaling up to other clinics.

### Study Component 3: Qualitative Study

#### Aim

Broadly, we aim to qualitatively assess:

1. The level of feasibility (implementation, acceptability, adoptability, practicality, and adaptability), sustainability, and scalability of IMPACTFrail’s core components and implementation strategies, and barriers and facilitators to their attainment.
2. Effectiveness of implementation strategies. This refers to the attribution of implementation strategies to attaining feasibility, sustainability, and scalability.

#### Study Sample and Data Source

Study participants will include older adult participants, clinic implementation leads who oversaw the planning and execution of IMPACTFrail, and frontline health care providers involved in the program’s core functions (doctors, nurses, care managers, care coordinators, allied health professionals, and support staff). We will conduct purposive sampling and sample sizes will be guided by the concept of sufficiency [51,52]. First, we will include older adults who participated in IMPACTFrail. We aim to interview a minimum of 3-4 older adults per clinic, with a total of at least 15 participants (approximately 10% of the targeted 36 recruited participants per clinic). We will sample the older adults using maximum variation for representation across demographic variables such as sex, ethnic groups, and age groups. Second, the qualitative study component will include clinic implementation leads who oversaw the planning and execution of IMPACTFrail and frontline health care providers involved in the program’s core functions (doctors, nurses, paraprofessional support staff). We anticipate only a small number of health care workers being involved in this small-scale pilot study. Hence, we will aim to sample all implementation leads at each clinic, estimated to range from 2 to 4 individuals per site. Additionally, we aim to sample frontline health care workers across every occupation who was involved across each central function of IMPACTFrail. Nurses and paraprofessional support staff in the qualitative study would need to have conducted IC screening for at least 5 older adults and doctors would need to conduct follow-up clinical assessments to assess intrinsic capacity deficits for at least 5 older adults. Interviews and focus groups with clinic implementation leads and frontline health care providers will be conducted in English, while interviews with older adults will be in either English or Chinese.

#### Study Procedure

As mentioned, both data collection and data analysis will be guided by the 4 feasibility criteria, the updated CFIR and its Outcomes addendum, TDF, and ERIC taxonomy [31,32,49]. We will expand CFIR’s Individual Characteristics domain by mapping determinants of capability, opportunity, and motivation to TDF domains [69,70]. The ERIC taxonomy informs the refinement of existing implementation strategies and selection of new strategies [32]. The semistructured topic guides elicit data that inform the 4 feasibility criteria, CFIR-based and TDF-based barriers and facilitators, and suggestions on new ERIC strategies. For example, for the criteria on implementation, we will obtain perspectives from implementers on the barriers and facilitators to delivering IMPACTFrail’s core components and implementation strategies (eg, mobilizing program champions, attending training), and perspectives on the extent that the strategies enabled the implementation of IMPACTFrail. Older adults will share about the barriers and facilitators of participating in IMPACTFrail and receiving implementation strategies (eg, patient educational materials) and the extent to which the strategies have facilitated their participation.

We aim to conduct 2 rounds of 14 interviews with clinic implementation leads across all 5 clinics. The first round of interviews will be conducted within the first 2 months of IMPACTFrail’s roll out. This will inform early implementation barriers to refine its delivery and inform additional support required. The second round of interviews will be conducted toward the last 4-5 months of the study and after the first 20-25 older adults have been recruited. This is to elicit their perspectives on how implementation has progressed, new barriers and facilitators that arose, and insights on improving the program. Additionally, we will conduct interviews or focus groups with frontline health care providers involved in delivering IMPACTFrail’s core functions. In the first instance, we will aim to conduct focus groups, as opposed to interviews, among the health care providers who worked as a team, if scheduling allows.

Deductive analysis will be guided by the frameworks and concurrent inductive analysis will reveal emergent barriers, facilitators, and themes. Two trained research team members will code verbatim transcripts independently and subsequently reconcile discrepancies. Framework analysis will be conducted [71]. This allows timely charting of data into a framework matrix, generation of themes by linking conceptually related codes, and directly addressing the feasibility criteria (Table 2). We will conduct a thematic analysis using data collected across all 3 regional health systems, as we anticipate cross-cutting barriers, facilitators and learning points across study sites. We will also identify contextual insights specific to each regional health system. This analytic approach allows us to synthesize cross-site patterns with a larger amount of data, while accounting for differences in organizational context, and directly address the feasibility of implementation across settings. A pragmatic approach of concurrent data collection and analysis will be adopted to ensure timely reporting of findings to clinic leadership and stakeholders to support the preparation of the scaled-up phase [72-75]. Microsoft Excel and NVivo 12 (QSR International) will be used for data management.

### Study Component 4: Cost Analysis

#### Aim

We aim to estimate the cost of developing and delivering the program and implementation strategies, with the purpose of informing the amount of resources required to sustain and to expand the program beyond the feasibility study.

#### Study Procedure and Data Source

First, we will compute developmental costs, which comprise (1) developing IMPACTFrail’s core functions, and (2) developing each implementation strategy. The expenditures on manpower (eg, health care providers involved in the co-development phase) and other expenses (materials, printing, etc) will be aggregated. Second, program costs will be derived from the costs of (1) delivering IMPACTFrail’s core components, and (2) operationalizing each implementation strategy. The expenditures on manpower, equipment, and operating expenses will be aggregated.

A time-driven, activity-based micro-costing approach will be used to estimate costs related to manpower resourcing [76,77]. Specifically, this will be derived from the unit cost multiplied by the intensity of use and duration of use. The unit cost will be estimated based on financial records or the standardized workforce unit cost for implementers obtained from the clinics. The intensity and duration of use will be derived from self-reported time spent for a select list of activities by implementers to deliver the program [78]. We will only use financial statements or costs self-reported by implementation leads to obtain unit costs on equipment and other operating expenses and their intensity of use.

Within each site, we will present the cost associated with (1) developing IMPACTFrail’s core functions, (2) developing each implementation strategy, (3) delivering IMPACTFrail’s core functions, (4) delivering each implementation strategy, (5) total developmental cost, (6) total program cost, and (7) total costs.

### Study Component 5: Program Description

We aim to produce written program descriptions across clinics guided by frameworks including the Template for Intervention Description and Replication (TIDieR) checklist or the Aims, Ingredients, Mechanism, and Delivery (AMID) framework [79,80]. The program descriptions will facilitate transparency and replicability.

### Study Component 6: Logic Model and Theory of Change

**Figure 2:**
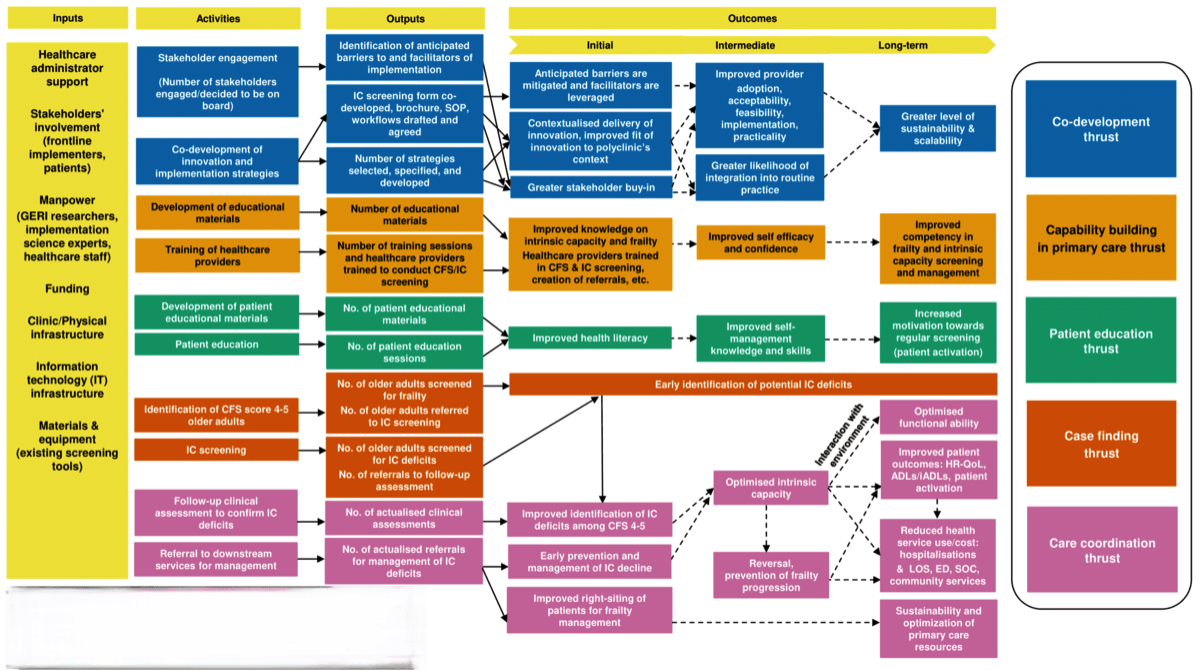
Initial logic model and theory of change. Bold arrow represents the link of an activity to outputs or outcomes. Broken arrow represents the link of an activity to outputs or outcomes. ADL: activity of daily living; CFS: Clinical Frailty Scale; ED: emergency department; GERI: Geriatric Education & Research Institute; HR-QoL: health-related quality of life; iADL: instrumental activity of daily living; IC: intrinsic capacity; LOS: length of stay; SOC: specialist outpatient clinic; SOP: standard operating procedures;.

The logic model and theory of change depict a visual representation of the resources, key activities, outputs, outcomes, and plausible causal pathways connecting them [81,82]. Figure 2 displays the initial theory of change. During this feasibility study, we will refine the logic model and theory of change. This updated logic model and theory of change will guide our understanding on the mechanisms of change and on the short to longer term health outcomes to evaluate in the future study.

### Triangulation of Findings

For each of the 4 feasibility criteria, the findings across study components will be presented and narratively triangulated (Table 2). Next, findings across all criteria will be narratively triangulated and assessed holistically to inform feasibility and readiness to scale. Briefly, for instance, under the criteria on implementation, we will triangulate the quantitative process indicators on completion rates of IC screening, along with CFIR-based barriers to participating perceived by older adults and barriers to implementing IC screening by health care providers. Under the criteria on practicality, we may triangulate the cost analysis of incorporating IC screening into clinic workflows, such as manpower and operating costs, CFIR-based barriers on resource allocation and management, and perspectives on sustainability. Under the criteria on adaptability, we will elicit if the barriers could be mitigated by implementation strategies and refinements to the program. A key feasibility assessment result that will suggest a “go” is at least 80% of the targeted recruitment at each of the participating clinics; that is, at least 29 of the targeted 36 older adults are recruited into the study and complete IC screening. This will demonstrate that the key novel core function of the program, which is to deliver IC screening of 6 health domains, is feasible. Importantly, we will be guided and informed by the findings across the multiple study components and methodologies. Each of the 3 regional health systems in Singapore will be engaged. Overall, we will evaluate the readiness to scale IC screening to more older adults at the 5 clinics and to additional new clinics across the 3 health care systems. Stakeholders will be engaged to review the findings and discuss readiness to scale, including research directors from the 3 health care systems, health care administrators (clinic leadership), and frontline health care providers. This process is aligned with the UK MRC Framework, FAID, and implementation research frameworks.

### Ethical Considerations

Ethical approval for this study was obtained from the NHG Domain Specific Review Board (ECOS Ref: 2024-3878). Written informed consent will be taken from all participants prior to their participation in the study. Older adult participants will receive SGD $30 (US $23.65) upon completion of informed consent-taking and IC screening, and SGD $50 (US $39.42) upon completion of the interview. All data collected will be anonymized and securely stored in a restricted-access network accessible only to the research team.

## Results

The study was funded in September 2024. Data collection for the feasibility testing of IMPACTFrail commenced in April 2025 and will conclude by March 2026. As of manuscript submission, only 98 participants have been recruited across 5 sites. Recruitment, data collection, and data analysis are still ongoing. The results are expected to be published in early 2027.

## Discussion

### Principal Findings

IMPACTFrail seeks to translate Singapore’s National Frailty Strategy and the WHO ICOPE framework by serving as an IC detection and management program for mildly frail older adults in primary care [16]. It contributes to a holistic approach to healthy aging by enabling early mitigation of frailty progression and optimizing functional ability. At a broader level, it is aligned with the WHO public health framework on healthy aging [7], strengthens Singapore’s contribution to the United Nations Decade of Healthy Ageing (2021-2030) [83], and complements national initiatives on preventative care for chronic conditions in primary care (eg, Healthier SG) and aging well in the community (eg, Age Well SG) [41,42].

The implementation and evaluation of the ICOPE framework in real-world practice is still in its nascence [17]. A systematic review reported that most studies have only used IC primarily as an outcome measure, rather than to inform intervention design [84]. The existing studies have focused on examining the prevalence of IC deficits or tool performance [85-87]. Implementation of the ICOPE programs in the community by early adopters in France and China demonstrated general acceptance of IC screening, but there are still gaps in understanding how to increase uptake and adherence among less activated older adults, and scale up beyond the feasibility testing sites that had more resources and stakeholder buy-in [19-21]. In Korea, Won [38] described the first small-scale ICOPE program implemented in primary care, contextualized to concurrently assess frailty and IC, using a 53-item screening approach. However, scaling up and effectiveness have not been reported.

In response, our adoption of a systematic and staged approach grounded in implementation science aims to accelerate the translation of Singapore’s National Frailty Strategy and the WHO ICOPE framework with high reach, fidelity, adoption, sustainability, and scalability. Moreover, embedding implementation considerations from the outset may strengthen the program’s design and delivery, hence improving its effectiveness, sustainability, and scalability in real-world settings. If the findings indicate limited effectiveness, the likelihood of confounding from poor implementation and fidelity is lower; conversely, effectiveness elicited under research study conditions tends to be better sustained in real-world settings [88-90]. A second key strength is the integration of complex intervention research and implementation research frameworks to guide the co-development of the program and implementation strategies, the feasibility assessment, and the qualitative study. This integrated approach enhances methodological rigor and increases the likelihood of successful and contextualized implementation of a complex intervention. Third, IMPACTFrail is co-developed from the outset in collaboration with multiple stakeholders across all 3 regional health systems in Singapore. This is imperative in facilitating scalability across more clinics within each cluster and reduces cross-cluster fragmentation of services. Fourth, our feasibility assessment is informed by published guidelines on conducting feasibility studies, precise mapping of study components to feasibility criteria, and adopting a mixed methods approach. This facilitates the depth and quality of insights through triangulation of findings.

There are several potential limitations. First, variations in eligibility criteria across clinics may limit the comparability of findings across sites and reduce generalizability. However, this reflects the real-world implementation context of IMPACTFrail, which is operationalized across three public health care clusters with distinct workflows, resources, and different population needs. As such, contextual adaptations guided by complex intervention research and implementation science frameworks are necessary. While this may limit generalizability, it generates insights to inform the future scale-up of IMPACTFrail and other similar programs in primary care. Second, findings may be less generalizable to settings beyond primary care and to older adults who are not mildly frail. However, insights related to common barriers, such as capability building, resource allocation and patient-level behaviors, will be applicable. We will also report how these insights are used to refine the delivery of the program’s core steps and implementation strategies to support future scale-up. Importantly, this article still serves to disseminate generalizable knowledge on the implementation research methodologies and approaches to co-develop an ICOPE-based intervention with multiple stakeholders from the outset, and the structure use of feasibility testing prior to a larger main study. Third, the feasibility study does not include a comparator arm. However, the primary aim is not to assess effectiveness but to apply implementation science methodologies that enhance the likelihood of sustainability and scalability of a complex intervention nationwide. Effectiveness outcomes will be evaluated in a subsequent hybrid effectiveness-implementation study [57,58], which will be statistically powered to evaluate impacts on patient outcomes (eg, function, quality of life), health care use, and cost effectiveness. Lastly, tracking actualization rates of health and social care services for managing IC deficits may be incomplete due to the absence of a common information technology system across public primary care, private specialist clinics, and selected community organizations. Nevertheless, referral actualization within the primary care setting (eg, memory clinic and health and wellness clinic) and most public specialist outpatient clinics will be captured and monitored.

### Conclusions

In summary, this study contributes to the literature by providing a detailed study protocol on the co-development and feasibility testing of a complex intervention to enhance transparency, fidelity, and replicability. It demonstrates the application of a systematic implementation science approach, the integration of complex intervention and implementation research frameworks, and a multimethods feasibility assessment to accelerate the translation of evidence to sustainable and scalable programs in real-world practice.

## Data Availability

Data sharing is not applicable to this article as no datasets were generated or analyzed during this study.

## Appendix

Multimedia Appendix 1 SPIRIT 2025 checklist of items to address in a randomized trial protocol.

Multimedia Appendix 2 World Health Organization integrated care for older people (WHO ICOPE) screening tool for submission.

## Abbreviations

AMID: Aims, Ingredients, Mechanism, and Delivery
CFIR: Consolidated Framework for Implementation Research
CFS: Clinical Frailty Scale
CGA: comprehensive geriatric assessment
COM-B: Capability, Opportunity, Motivation, Behavior
ERIC: Expert Recommendations for Implementing Change
FAID: Framework of Actions for Intervention Development
IC: intrinsic capacity
ICOPE: integrated care for older people
IMPACTFrail: Intrinsic Capacity Promotion in Primary Care for the Frail
MOH: Ministry of Health
MRC: Medical Research Council
SPIRIT: Standard Protocol Items: Recommendations for Interventional Trials
TDF: Theoretical Domains Framework
TIDieR: Template for Intervention Description and Replication
WHO: World Health Organization
