# Supplementary material for: Development and Feasibility Assessment of an Intrinsic Capacity Program in Primary Care: Protocol for an Implementation Science Approach
Source: JMIR Res Protoc. 2026 Feb 2;15:e84257. doi: 10.2196/84257 (PMC12910274; doi:10.2196/84257)
Supplement: Multimedia Appendix 1 [file resprot_v15i1e84257_app1.pdf]

Supplementary Appendix

Supplementary Appendix File 1 SPIRIT 2025 Checklist of Items to Address in a Randomised Trial Protocol

Section/topic	No.	SPIRIT 2025 checklist item description	Reported section/remark
**Administrative information**			
Title and structured summary	1a	Title stating the trial design, population, and interventions, with identification as a protocol	Development and feasibility assessment of an intrinsic capacity screening management intervention for older adults in primary care: A Protocol
	1b	Structured summary of trial design and methods, including items from the World Health Organization Trial Registration Data Set	The study is registered on ClinicalTrials.gov (ID NCT06753643) on 22 December 2024. https://clinicaltrials.gov/study/NCT06753643?term=NCT06753643&rank=1
Protocol version	2	Version date and identifier	Version 1. ID NCT06753643.
Roles and responsibilities	3a	Names, affiliations, and roles of protocol contributors	Refer to *Author contributions statement*.
	3b	Name and contact information for the trial sponsor	Geriatric Education & Research Institute, Singapore<br>Email: enquiry@geri.com.sg
	3c	Role of trial sponsor and funders in design, conduct, analysis, and reporting of trial; including any authority over these activities	Trial sponsor and funders have no role in design, conduct, analysis, and reporting of trial.
	3d	Composition, roles, and responsibilities of the coordinating site, steering committee, endpoint adjudication committee, data management team, and other individuals or groups overseeing the trial, if applicable	Implementation leads at each of the five clinics oversee the delivery of the programme and strategies and coordinate data flow between the clinic and researchers.<br><br>The steering committee comprises of three research directors who will review findings from the feasibility study to assess readiness for progression to the larger-scale study.
**Open science**			

Trial registration	4	Name of trial registry, identifying number (with URL), and date of registration. If not yet registered, name of intended registry	ClinicalTrials.gov ID NCT06753643. Registered on 22 December 2024. https://clinicaltrials.gov/study/NCT06753643?term=NCT06753643&rank=1
Protocol and statistical analysis plan	5	Where the trial protocol and statistical analysis plan can be accessed	Refer to *Objective 2: Multi-site feasibility study (12 months)*, with detailed justification and explanation of study procedures and analysis plan for each study component.
Data sharing	6	Where and how the individual de-identified participant data (including data dictionary), statistical code, and any other materials will be accessible	The Principal Investigator will oversee the data sharing process.<br><br>All study team members will be given access to the de-identified data sets. Hardcopy documents with identifiable information will be stored in a secure and locked cabinet accessible only to the study team.<br><br>The data will not be shared with external parties outside the study team because we wish to protect the privacy rights of the participants in accordance with the informed consent of this study. Request to access the data should be directed to the Principal Investigator.
Funding and conflicts of interest	7a	Sources of funding and other support (e.g., supply of drugs)	This study is funded by the Geriatric Education & Research Institute (GERI) Intramural Grant (Project Reference: GERI1637).
	7b	Financial and other conflicts of interest for principal investigators and steering committee members	All authors and steering committee members had no competing interests.
Dissemination policy	8	Plans to communicate trial results to participants, healthcare professionals, the public, and other relevant groups (e.g., reporting in trial registry, plain language summary, publication)	The study team will disseminate study findingsvia publications in international peer-reviewed journals, research briefs, conferences, and knowledge exchange platforms
**Introduction**			

Background and rationale	9a	Scientific background and rationale, including summary of relevant studies (published and unpublished) examining benefits and harms for each intervention	Refer to *Introduction.*
	9b	Explanation for choice of comparator	There is no comparator arm. Refer to *Study Design*.
Objectives	10	Specific objectives related to benefits and harms	Refer to *Introduction: Study aims and hypotheses*.
**Methods: Patient and public involvement, trial design**			
Patient and public involvement	11	Details of, or plans for, patient or public involvement in the design, conduct, and reporting of the trial	Refer to *Methods: Objective 1: Collaborative development of IMPACTFrail and implementation strategies (6 months) – Study procedure*. The team conducted six months of informal stakeholder engagement sessions with clinic healthcare administrators, frontline healthcare providers (doctors, nurses, allied health professionals), and older adults who were existing patients of the clinics. The engagement aimed to explore awareness of frailty and IC, elicit anticipated barriers and facilitators of implementing or participating in IMPACTFrail, and understand the contextual operational and logistical constraints at each clinic. This study builds on the information from the stakeholder engagement.
Trial design	12	Description of trial design including type of trial (e.g., parallel group, crossover), allocation ratio, and framework (e.g., superiority, equivalence, non-inferiority, exploratory)	Refer to *Methods: Objective 2: Multi-site feasibility study (12 months)*. This is a single-arm multi-site feasibility study. The study has no comparator arm or pre-post design, because we do not examine effectiveness. We focus on a small-scale feasibility assessment, in preparation for a subsequent hybrid effectiveness-implementation study that will be statistically powered.
**Methods: Participants, interventions, and outcomes**			

Trial setting	13	Settings (e.g., community, hospital) and locations (e.g., countries, sites) where the trial will be conducted	Refer to *Methods: Objective 2: Multi-site feasibility study (12 months) – Study design*.
Eligibility criteria	14a	Eligibility criteria for participants	Refer to *Methods: Objective 2: Multi-site feasibility study (12 months) – Eligibility criteria and sample size.*
	14b	If applicable, eligibility criteria for sites and for individuals who will deliver the interventions (e.g., surgeons, physiotherapists)	Not applicable
Intervention and comparator	15a	Intervention and comparator with sufficient details allow replication including how, when, and by whom they will be administered. If relevant, where additional materials describing the intervention and comparator (e.g., intervention manual) can be accessed	Refer to *Introduction: INTRINSIC CAPACITY PROMOTION IN PRIMARY CARE FOR THE FRAIL (IMPACTFrial) PROGRAMME.*<br><br>Refer to *Methods: Refer to Objective 2: Multi-site feasibility study (12 months)*. This is a single-arm multi-site feasibility study without no comparator arm or pre-post design, as it focuses on small-scale feasibility rather than effectiveness. This study serves as the preparation for a subsequent, statistically powered hybrid effectiveness-implementation study.
	15b	Criteria for discontinuing or modifying allocated intervention/comparator for a trial participant (e.g., drug dose change in response to harms, participant request, or improving/worsening disease)	This is a healthcare screening programme with very low to minimal risk of harm. Participants can discontinue programme participation at any point in time at their request.
	15c	Strategies to improve adherence to intervention/comparator protocols, if applicable, and any procedures for monitoring adherence (e.g., drug tablet return, sessions attended)	Refer to *Methods: Objective 2: Multi-site feasibility study (12 months) - Study component 1: Quantitative process indicators on care processes*. A list of process indicators is summarised in Table 3.<br><br>Process indicators will be collected monthly to track implementation progress and monitor adherence. by trained frontline healthcare providers and research assistants/clinical research coordinators

			from the electronic medical record system, and verified by designated study team members.<br><br>Strategies such as phone calls or text reminders for appointments will be used to increase intervention adherence.
	15d	Concomitant care that is permitted or prohibited during the trial	All standard care, such as management of chronic diseases, is permitted. No clinical care will be prohibited.
Outcomes	16	Primary and secondary outcomes, including the specific measurement variable (e.g., systolic blood pressure), analysis metric (e.g., change from baseline, final value, time to event), method of aggregation (e.g., median, proportion), and time point for each outcome	We have two objectives: (1) to collaboratively develop IMPACTFrail's core components and implementation strategies; and (2) to conduct a multi-site feasibility study.<br><br>For the first objective, we hypothesise a successful co-development of the programme's core components and implementation strategies. Examples of specific output from the co-development include IC screening questions adapted to the clinics' contexts, and specification of strategies implementation plan.<br><br>For the second objective, we will use four outcomes to assess feasibility i.e., **(1) implementation** (How well was it implemented as intended?), **(2) acceptability** (Is it satisfactory to deliverers and recipients?), **(3) practicality** (How practical is it to carry out?), and **(4) adaptability** (Are there solutions to the barriers we encounter?).
Harms	17	How harms are defined and will be assessed (e.g., systematically, non-systematically)	The programme has less than minimal risk to participants and we do not anticipate adverse events directly from this study. However,

			participants may expose to physical, psychological or social risks.<br>• **Physical risk:** participants may injure themselves if they carry out the physical components of the follow-up assessments (e.g. chair rise test) in an improper manner. **The following steps will be undertaken to minimise the risk:** Participants will be assessed by healthcare professionals who are trained in participant safety. The physical tests will be conducted for one participant at a time to ensure the participant's safety. The healthcare professional may stop the participant from continuing with the physical assessments if they deem it to be in the participant's best interest. Participants may also stop the physical assessments if they feel unwell or are unable to complete them.<br><br>• **Psychological risk:** there is a risk that the participants may feel uncomfortable answering some questions of the focus groups and interviews. **To minimise this psychological risk,** participants can choose not to answer questions that they feel uncomfortable with.<br><br>• **Legal risk:** the legal risks are expected to be minimal but may come from a breach of confidentiality of data as participants will provide identifiable information. **For identifiable data collected on hardcopy documents** (e.g. identification log, informed consent, acknowledgement of tokens of

			appreciation), these will be kept by the respective study sites or GERI under lock and key in a secured location. Softcopy data (e.g. audio recordings) will be password protected and stored in the study sites' or GERI's access-controlled cloud storage platforms. Data will be de-identified before analysis. Any data to be analysed will be password protected and only the research team will have access to the data.
Participant timeline	18	Time schedule of enrollment, interventions (including any run-ins and washouts), assessments, and visits for participants. A schematic diagram is highly recommended (see Figure)	Refer to *Introduction (Programme description): INTRINSIC CAPACITY PROMOTION IN PRIMARY CARE FOR THE FRAIL (IMPACTFrial) PROGRAMME*. Participants will first undergo frailty screening using the Clinical Frailty Scale (CFS), and those identified as mildly frail (CFS 4 or 5) will proceed to a six-domain intrinsic capacity (IC) screening after inform consent is taken. Participants with IC deficits will then be scheduled for follow-up clinical assessments immediately after screening. Based on the assessment, patients will be referred to health and/or social services to manage their IC deficits.<br><br>For the qualitative research component, refer to *Methods: Multi-site feasibility study (12 months) – Study component 3: Qualitative study*. Interviews with clinic implementation leads across all 5 clinics within the first two months of the programme roll out will be conducted by trained study team members. Participants are anticipated to spend about 90 to 120 minutes on the interviews or focus groups.

Sample size	19	How sample size was determined, including all assumptions supporting the sample size calculation	Refer to *Methods: Objective 2: Multi-site feasibility study (12 months) – Eligibility criteria and sample size*.
Recruitment	20	Strategies for achieving adequate participant enrollment to reach target sample size	Refer to *Methods: Objective 2: Multi-site feasibility study (12 months) – Eligibility criteria and sample size*.<br><br>Strategies such as phone calls or text reminders for appointments will be used to increase intervention adherence.<br><br>A research brochure outlining the programme, procedures, associated cost, potentials benefits and harms will be provided to patients, along with a script to support communication by healthcare providers during recruitment.
**Methods: Assignment of interventions**			
Randomization:			
Sequence generation	21a	Who will generate the random allocation sequence and the method used	Not applicable
	21b	Type of randomization (simple or restricted) and details of any factors for stratification. To reduce predictability of a random sequence, other details of any planned restriction (e.g., blocking) should be provided in a separate document that is unavailable to those who enroll participants or assign interventions	Not applicable
Allocation concealment mechanism	22	Mechanism used to implement the random allocation sequence (e.g., central computer/telephone; sequentially numbered, opaque, sealed containers), describing any steps to conceal the sequence until interventions are assigned	Not applicable
Implementation	23	Whether the personnel who will enroll and those who will assign participants to the interventions will have access to the random allocation sequence	Not applicable

Blinding	24a	Who will be blinded after assignment to interventions (e.g., participants, care providers, outcome assessors, data analysts)	Not applicable
	24b	If blinded, how blinding will be achieved and description of the similarity of interventions	Not applicable
	24c	If blinded, circumstances under which unblinding is permissible, and procedure for revealing a participant's allocated intervention during the trial	Not applicable
**Methods: Data collection, management, and analysis**			
Data collection methods	25a	Plans for assessment and collection of trial data, including any related processes to promote data quality (e.g., duplicate measurements, training of assessors) and a description of trial instruments (e.g., questionnaires, laboratory tests) along with their reliability and validity, if known. Reference to where data collection forms can be accessed, if not in the protocol	Data will be collected by trained frontline healthcare providers and research assistants/clinical research coordinators from the electronic medical record system. To ensure data quality, all data entered will be checked by the study team for consistency and completeness.
	25b	Plans to promote participant retention and complete follow-up, including list of any outcome data to be collected for participants who discontinue or deviate from intervention protocols	Refer to *Methods: Objective 2: Multi-site feasibility study (12 months) - Eligibility criteria and sample size*. The target recruitment numbers of n=36 participant per site account for a 20% dropout.<br><br>Strategies such as phone calls or text reminders for appointments will be used to increase intervention adherence.<br><br>Participants may choose to drop out at any point and/or investigators may withdraw participants from the study for safety reasons. Reasons for loss to follow-up will be documented, and data collected until the point of drop out will be used in analysis.
Data management	26	Plans for data entry, coding, security, and storage, including any related processes to promote data quality (e.g., double data entry; range checks for data values). Reference to where details of data management procedures can be accessed, if not in the protocol	For the screenings, participant data will be entered by trained frontline healthcare providers. Research assistants/clinical research coordinators in the clinics will extract data from participants' electronic

			medical records and will be responsible for de-identifying the data.<br><br>To ensure data quality and integrity, all data will be verified by designated study team members for consistency and completeness. Principal Investigator and site Principal Investigators have close oversight on integrity.<br><br>For qualitative research, data will be collected by trained study team members through interviews or focus groups. All data will be de-identified, password-protected and stored in GERI's secured cloud storage system and corporate computers.
Statistical methods	27a	Statistical methods used to compare groups for primary and secondary outcomes, including harms	Refer to *Methods: Objective 2: Multi-site feasibility study (12 months)*.
	27b	Definition of who will be included in each analysis (e.g., all randomized participants), and in which group	Not applicable
	27c	How missing data will be handled in the analysis	Not applicable
	27d	Methods for any additional analyses (e.g., subgroup and sensitivity analyses)	Not applicable
**Methods: Monitoring**			
Data monitoring committee	28a	Composition of data monitoring committee (DMC); summary of its role and reporting structure; statement of whether it is independent from the sponsor and funder; conflicts of interest and reference to where further details about its charter can be found, if not in the protocol. Alternatively, an explanation of why a DMC is not needed	There will be no independent Data Monitoring Committee, as the intervention has less than minimal risk and we do not anticipate adverse events directly from this study. The programme conduct will be monitored by the study team and audited by the NHG DSRB.
	28b	Explanation of any interim analyses and stopping guidelines, including who will have access to these interim results and make the final decision to terminate the trial	This is a healthcare screening programme with very low to minimal risk of harm. Participants can discontinue programme participation at any point in time at their request.

Trial monitoring	29	Frequency and procedures for monitoring trial conduct. If there is no monitoring, give explanation	Refer to *Methods: Objective 2: Multi-site feasibility study (12 months) - Study component 1: Quantitative process indicators on care processes*.<br><br>Quantitative process indicators to measure the processes of care and track the fidelity of implementation strategies will be collected and documented bi-weekly to track implementation and monitor progress. To ensure data quality, all data collected will be verified by the designated study team for consistency and completeness.
**Ethics**			
Research ethics approval	30	Plans for seeking research ethics committee/institutional review board approval	Refer to Declarations: Ethics approval
Protocol amendments	31	Plans for communicating important protocol modifications to relevant parties	Protocol modifications that affect the study conduct, potential risks, benefits and safety of the participants will require a formal amendment of the protocol. Any amendments to the protocol described in this paper will be approved by the by the NHG DSRB and agreed on by the Sponsor.
Consent or assent	32a	Who will obtain informed consent or assent from potential trial participants or authorized proxies, and how	Informed consent will be taken by trained study team members. Participants will be given sufficient time to read the informed consent document, clarify and ask questions. If eligible participants agree to be enrolled, written informed consent will be taken.
	32b	Additional consent provisions for collection and use of participant data and biological specimens in ancillary studies, if applicable	Not applicable
Confidentiality	33	How personal information about potential and enrolled participants will be collected, shared, and maintained in order to protect confidentiality before, during, and after the trial	Personal information will be de-identified through the use of a unique participant study number assigned to the participant. All the data will be password-protected and stored in GERI's secured cloud storage system and corporate computers. Access to this information will be restricted to the

			study team only. As per NHG DSRB requirements, all data will be maintained for a period of 6 years after completion of the study.
Ancillary and post-trial care	34	Provisions, if any, for ancillary and post-trial care, and for compensation to those who suffer harm from trial participation	If participants follow the directions of the study team and they are physically injured due to the procedure given under the plan of this study, the Geriatric Education & Research Institute will compensate the medical expenses for the treatment of that injury.

HrÃ³bjartsson A, Boutron I, Hopewell S, Moher D, Schulz K F, Collins G S et al. SPIRIT 2025 explanation and elaboration: updated guideline for protocols of randomised trials *BMJ* 2025; 389 :e081660 doi:10.1136/bmj-2024-081660
