# Supplementary material for: Development and Feasibility Assessment of an Intrinsic Capacity Program in Primary Care: Protocol for an Implementation Science Approach
Source: JMIR Res Protoc. 2026 Feb 2;15:e84257. doi: 10.2196/84257 (PMC12910274; doi:10.2196/84257)
Supplement: Multimedia Appendix 2 [file resprot_v15i1e84257_app2.pdf]

**Supplementary Appendix File 2.** WHO ICOPE Screening Tool

| Priority conditions associated with declines in intrinsic capacity | Tests | Assess fully any domain with a checked circle |
|---|---|---|
| **COGNITIVE DECLINE** | 1. Remember three words: flower, door, rice (for example) | |
| | 2. Orientation in time and space: What is the full date today? Where are you now (home, clinic, etc)? | ○ Wrong to either question or does not know |
| | 3. Recalls the three words? | ○ Cannot recall all three words |
| **LIMITED MOBILITY** | Chair rise test: Rise from chair five times without using arms. Did the person complete five chair rises within 14 seconds? | ○ No |
| **MALNUTRITION** | 1. Weight loss: Have you unintentionally lost more than 3 kg over the last three months? | ○ Yes |
| | 2. Appetite loss: Have you experienced loss of appetite? | ○ Yes |
| **VISUAL IMPAIRMENT** | Do you have any problems with your eyes: difficulties in seeing far, reading, eye diseases or currently under medical treatment (e.g. diabetes, high blood pressure)? | ○ Yes |
| **HEARING LOSS** | Hears whispers (whisper test) ***or*** Screening audiometry result is 35 dB or less ***or*** Passes automated app-based digits-in-noise test | ○ Fail |
| **DEPRESSIVE SYMPTOMS** | Over the past two weeks, have you been bothered by – feeling down, depressed or hopeless? | ○ Yes |
| | – little interest or pleasure in doing things? | ○ Yes |

World Health Organization. (2019). *Integrated care for older people (ICOPE): guidance for person-centred assessment and pathways in primary care*. https://www.who.int/publications/i/item/WHO-FWC-ALC-19.1
